# Protein sequence-similarity search acceleration using a heuristic algorithm with a sensitive matrix

**DOI:** 10.1007/s10969-016-9210-4

**Published:** 2017-01-12

**Authors:** Kyungtaek Lim, Kazunori D. Yamada, Martin C. Frith, Kentaro Tomii

**Affiliations:** 1 Artificial Intelligence Research Center, National Institute of Advanced Industrial Science and Technology (AIST), 2-4-7 Aomi, Koto-ku, Tokyo, 135-0064 Japan; 2 Graduate School of Information Sciences, Tohoku University, 6-3-9 Aramaki-Aza-Aoba, Aoba-ku, Sendai, 980-8579 Japan; 3 Department of Computational Biology and Medical Sciences, University of Tokyo, 5-1-5 Kashiwa-no-ha, Kashiwa, Chiba 227-8561 Japan; 4 Biotechnology Research Institute for Drug Discovery, National Institute of Advanced Industrial Science and Technology (AIST), 2-4-7 Aomi, Koto-ku, Tokyo, 135-0064 Japan

**Keywords:** Amino acid substitution matrix, Homology detection, Alignment quality

## Abstract

Protein database search for public databases is a fundamental step in the target selection of proteins in structural and functional genomics and also for inferring protein structure, function, and evolution. Most database search methods employ amino acid substitution matrices to score amino acid pairs. The choice of substitution matrix strongly affects homology detection performance. We earlier proposed a substitution matrix named MIQS that was optimized for distant protein homology search. Herein we further evaluate MIQS in combination with LAST, a heuristic and fast database search tool with a tunable sensitivity parameter *m*, where larger *m* denotes higher sensitivity. Results show that MIQS substantially improves the homology detection and alignment quality performance of LAST across diverse *m* parameters. Against a protein database consisting of approximately 15 million sequences, LAST with *m* = 10^5^ achieves better homology detection performance than BLASTP, and completes the search 20 times faster. Compared to the most sensitive existing methods being used today, CS-BLAST and SSEARCH, LAST with MIQS and *m* = 10^6^ shows comparable homology detection performance at 2.0 and 3.9 times greater speed, respectively. Results demonstrate that MIQS-powered LAST is a time-efficient method for sensitive and accurate homology search.

**Electronic supplementary material:**

The online version of this article (doi:10.1007/s10969-016-9210-4) contains supplementary material, which is available to authorized users.

## Introduction

Protein homologs are likely to have similar structures, performing similar functions. Therefore, searching for protein homologs with known structures and functions is generally the first and most important step for selecting proteins for study and sample production, and for target selection in the field of structural and functional genomics. It is also a necessary task for biological and functional annotation in modern biology. Database search methods such as BLASTP [1] and SSEARCH [2] have been widely used for this purpose.

Considering the relative closeness between amino acids can help to enhance the sensitivity of database search methods. Amino acids are classifiable based on chemical properties stemming from their side chains, suggesting that substitutions between amino acid pairs occur at distinct rates according to similarity in their chemical properties. In turn, substitution probabilities presumably reflect relative similarities between amino acids. Many efforts have been undertaken to deduce amino acid substitution probabilities from a collection of protein sequences. These probabilities have been converted to residue pair scores, so that high sums of scores between two aligned sequences are useful as a measure of homology estimation. A 20 × 20 matrix consisting of scores of all amino acid pairs is called an amino acid substitution/scoring matrix. Classical substitution matrices such as PAM [3] and BLOSUM [4] are still dominant choices for homology search.

Many other substitution matrices have been proposed along with claims of superior performances. For example, some attempts have been undertaken to derive optimized matrices in terms of homolog discrimination performance [5–7] and alignment accuracy [8]. Maintaining the structural integrity of proteins is a fundamental constraint of amino acid substitution. Therefore, several earlier studies have been conducted to generate structure-dependent matrices [9–11]. Nevertheless, the use of structure-dependent matrices is restricted to proteins with structural information. One line of research has pursued incorporation of the sequence context into homology searches. Deviating from the form of substitution matrix, CS-BLAST deals with substitution probabilities in the form of a sequence profile computed based on nearby sequence context, by which significant sensitivity enhancement was achieved [12]. Implementation of non-standard context-specific methods in existing database search methods is not trivial. Therefore, inferring a better standard substitution matrix is expected to have a much broader impact on the database search technologies. We earlier proposed a highly sensitive matrix, which we call MIQS, by exploring the principal component subspace of classical substitution matrices, based on the postulation that there might be a chance to obtain better matrices for detecting distantly related proteins in the space around classical substitution matrices [13]. In that study, 990 points (=matrices) in the space were tested for their performance at remote homology detection to determine the optimal matrix, which was designated MIQS. We demonstrated that its application to SSEARCH achieved the highest level of homology detection performance among pairwise aligners [13].

Although SSEARCH is a highly performing database search method with respect to detection sensitivity, its time complexity is *O*(*mn*), where *m* and *n* are residue lengths of sequences to be compared. Because publicly available protein sequence data are increasing exponentially, database search method speeds are becoming increasingly important. For a more rapid database search, heuristic methods such as BLASTP and similar methods have been developed. Many heuristic methods first find short sequence matches (called seeds) to start alignment from, where longer seeds save time but decrease the detection sensitivity. In recent years, a fast aligner, LAST, which uses a suffix array of the target sequence(s) for finding ‘adaptive’ seeds, has been devised. LAST [14] can alleviate the tradeoff between time and sensitivity using the adaptive seed approach, where every seed is chosen not by a fixed length but by its frequency in the target database. LAST’s sensitivity is adjustable by a parameter *m*, which denotes the seed frequency threshold, i.e., selected seeds occur *m* or fewer times in the library database.

Actually, MIQS has not been tested for heuristic aligners, but only for the rigorous dynamic programming method (SSEARCH). Consequently, in this study, by application of MIQS to LAST with variation of the *m* parameter as a first trial, we demonstrate that it can achieve faster searching than rigorous dynamic programming methods, while maintaining comparable sensitivity. We also compare LAST to existing sensitive competitors to ascertain their potential as a remote protein homolog search method. The use of MIQS is shown to enhance LAST performance considerably across varying *m*. Moreover, LAST performance is dominant over BLASTP with respect to both sensitivity and time. LAST with MIQS is time-efficient compared to the most sensitive of existing methods: SSEARCH and CS-BLAST.

## Materials and methods

### Benchmark datasets

For benchmarking database search and alignment methods, databases of pre-classified homologs such as SCOP [15] and CATH [16] are useful. To evaluate methods for homology detection performances, we use two datasets that were used in our previous study [13]. From the SCOP 1.75 release, we obtained a non-redundant set of 7074 proteins, which was provided by the ASTRAL compendium [17] (SCOP20). The sequence identities between them are no more than 20%. SCOP20 was further divided into training (*n* = 3537) and validation (*n* = 3537) sets, which are available from our web site, http://csas.cbrc.jp/Ssearch/benchmark/. We refer to the validation set as *SCOP20 validation*, and used it for evaluating homology detection performances. Other datasets used for comparing detection performance are the CATH20-SCOP benchmark set [13], which is also available from our web site. It includes protein domain sequences (*n* = 1754) derived from CATH ver. 3.5.0, except those in the SCOP database, filtered using a maximum sequence identity of 20%.

The UniProt server provides the UniRef series that comprise representative sequences, each of which was chosen from a cluster consisting of sequences having more than a certain sequence identity [18]. For example, UniRef50 includes representative sequences from sequence groups clustered using a sequence identity of 50%. UniRef50 (15,327,814 sequences) was downloaded from ftp://ftp.uniprot.org/pub/databases/uniprot/uniref/uniref50/ on Oct 30, 2015. SCOP20 validation and UniRef50 were merged into UniRef50+. By searching for homologs of SCOP20 validation sequences in UniRef50+, database search methods were examined with a larger dataset to evaluate their performances and to assess appropriate options of LAST in more realistic situations. For simplicity, we considered only sequences from SCOP20 as positives. We ignored sequences from UniRef50 in the benchmark with UniRef50+.

To evaluate the alignment quality of each method, we used the subset of CATH20-SCOP benchmark set as in our previous study. We selected up to ten domain pairs randomly from each family in the CATH20-SCOP set and aligned each pair using DaliLite [19]. Alignments with Z-scores >2 generated by DaliLite were used as reference alignments. Thereby, we obtained reference alignments of 588 pairs from 670 domains. We compared sequence alignments generated by each method with the structural alignments generated by DaliLite.

### Alignment/search programs

We evaluated four database search methods. All were local aligners: one was from methods based on rigorous dynamic programming (SSEARCH 36.3.7b); the other three were from heuristic methods (BLASTP 2.2.27+, CS-BLAST 2.2.3, and LAST 638). We used default settings for BLASTP and CS-BLAST. We tested them with both BLOSUM62 and MIQS for SSEARCH and LAST. When we apply MIQS, we use gap penalties of −10 for open and −2 for extension for SSEARCH, and gap penalties of −13 and −2 for LAST. Gap penalties of −13 and −2 are the default settings of LAST with MIQS. Those values are sufficient to reduce overextended alignments, according to calibration with FLANK [20]. In LAST, we can control a tradeoff between speed and sensitivity through the −*m* option. This option designates the rareness limit for initial matches. The default value for this option is ten, meaning that selected seeds occur no more than ten times in the library database. Increasing this value makes LAST more sensitive but slower. We examined 10^2^, 10^3^, 10^4^, 10^5^, and 10^6^ as this value for the option to elucidate appropriate settings.

### Computational resource usage benchmark

Calculations for computational resource usage comparison were executed using a 2.70 GHz processor (Xeon(R) CPU E5-2680; Intel Corp.) in a Linux environment. The CPU time was measured using the *time* command. Maximum memory usage for each program was measured using the *qacct* command of the Sun Grid Engine.

## Results

### Homology detection performance comparison

Homolog detection is the key feature of database search methods. Structural classification of proteins (SCOP) and CATH databases comprise classified protein homologs with known structure. They have often been used for the evaluation of homology detection performance. The *SCOP20 validation set* (*n* = 3537) and CATH20-SCOP (*n* = 1754), consisting of protein sequences with pairwise similarity of no more than 20% was established previously for distant homology detection benchmarks (see “Materials and methods” section).

All-against-all search of the SCOP20 validation set permits the evaluation of database search performance for identification of distantly related proteins, i.e., homologs with <20% sequence identity. For a realistic database search benchmark, we constructed an expanded library dataset (UniRef50+), which includes the UniRef50 database (15,327,814 sequences) and the *SCOP20 validation set*. We submitted 2738 sequences, excluding 799 proteins (see below), of *SCOP20 validation* as query sequences against UniRef50+. We then examined hits from SCOP20 validation. When multiple hits were obtained for a single target protein, only the most significant one (with the lowest E-value) was chosen.

In this study, hits from the same SCOP superfamily classification for a query protein are regarded as true positives (TPs). Those from a different SCOP fold classification are labeled as false positives (FPs). Domains in the same fold might have a homologous relation (albeit more distant). Therefore, different superfamily hits from the same fold are defined as neither TPs nor FPs. There are arguably homology relations among some SCOP classifications even across folds. Thus, detection performance evaluation was also carried out, excluding FPs due to those putative homologous relations from the FP count according to the rule set by Julian Gough (JG) (http://www.supfam.org/SUPERFAMILY/ruleset.html) [21] as described in an earlier report [22, 23], where SCOP classifications with putative homologous relations are defined.

The ROC curve, which is a widely recognized mode of performance evaluation, draws TP and FP counts as a certain threshold varies, where a larger area under the ROC curve represents better performance. For each method, an ROC curve is drawn using the expected value (E-value) as the threshold across 2738 homology searches (here, we ignored 799 queries with no TPs except for themselves), where TP and FP counts are weighted by 1/(number of other homologs that belong to the query superfamily in *SCOP20 validation*) to prevent the bias from larger protein superfamilies from the ROC curve trend [12].

**Fig. 1:**
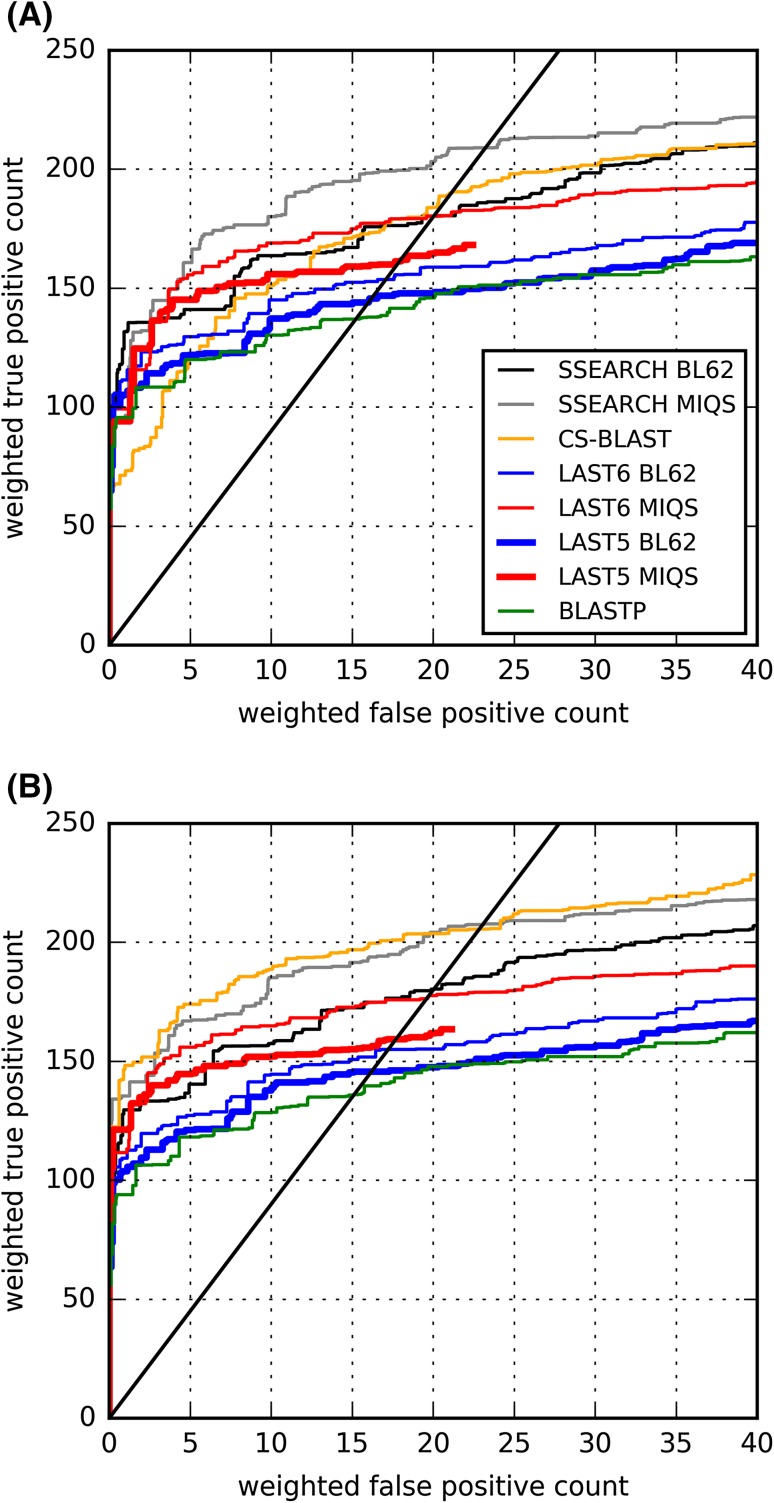
Superfamily level homology detection benchmark across database searches of the SCOP20 validation sequences against UniRef50+. ROC plot for weighted FP versus weighted TP counts up to particular E-values. Each FP or TP is weighted by 1/(number of the other domains in the query superfamily). Some FPs are ignored according to the JG standard in (**b**) but not in (**a**). *Solid black line* represents FDR = 10%. See “Results” section for additional details

The ROC plot in Fig. 1a shows that increasing *m* yields improved performance of LAST, as expected. Using BLOSUM62 (the default matrix of LAST), LAST with *m* = 10^5^ (hereinafter, LAST5) is able to detect 144 weighted TPs (wTPs), whereas LAST with *m* = 10^6^ (hereinafter, LAST6) detects 153.7 wTPs until a false discovery rate (FDR) of 10%. LAST5 exceeds BLASTP (wTP = 137 at FDR = 10%) in this benchmark. The application of MIQS improves LAST’s detection performances across both *m* values, compared with BLOSUM62. The performance of LAST6 with MIQS (wTP = 180.3 at FDR = 10%) is comparable to that of SSEARCH with BLOSUM62 (wTP = 180.3 at FDR = 10%) and is slightly less than that of CS-BLAST (wTP = 190 at FDR = 10%). As described earlier [13], MIQS also enhances SSEARCH performance, yielding the highest performance among those tested. Figure 1b presents the ROC plot as shown in Fig. 1a but with the Julian Gough (JG) standard. The curve trends closely resemble the non-JG standard version with the exception of CS-BLAST. CS-BLAST is the only method that shows a substantial ROC performance boost using the JG standard, surpassing the performance of SSEARCH with MIQS, though the performance of SSEARCH with MIQS is comparable to that of CS-BLAST at FDR = 10%. The relative performance of CS-BLAST in CATH20-SCOP is consistent with that in the *SCOP20 validation* benchmark without the JG standard. The performance boost only for CS-BLAST is remarkable, presumably because it was trained with a similar definition to the JG standard [22]. Regarding the larger library, we confirmed that we were able to obtain almost identical ROC curves in all-against-all comparisons only using *SCOP20 validation*, except for *m* parameters. LAST6-against-UniRef50+ is approximately equivalent to LAST4-against-SCOP20 validation (Fig. S1). We learned that larger *m* values should be used for the larger library.

**Fig. 2:**
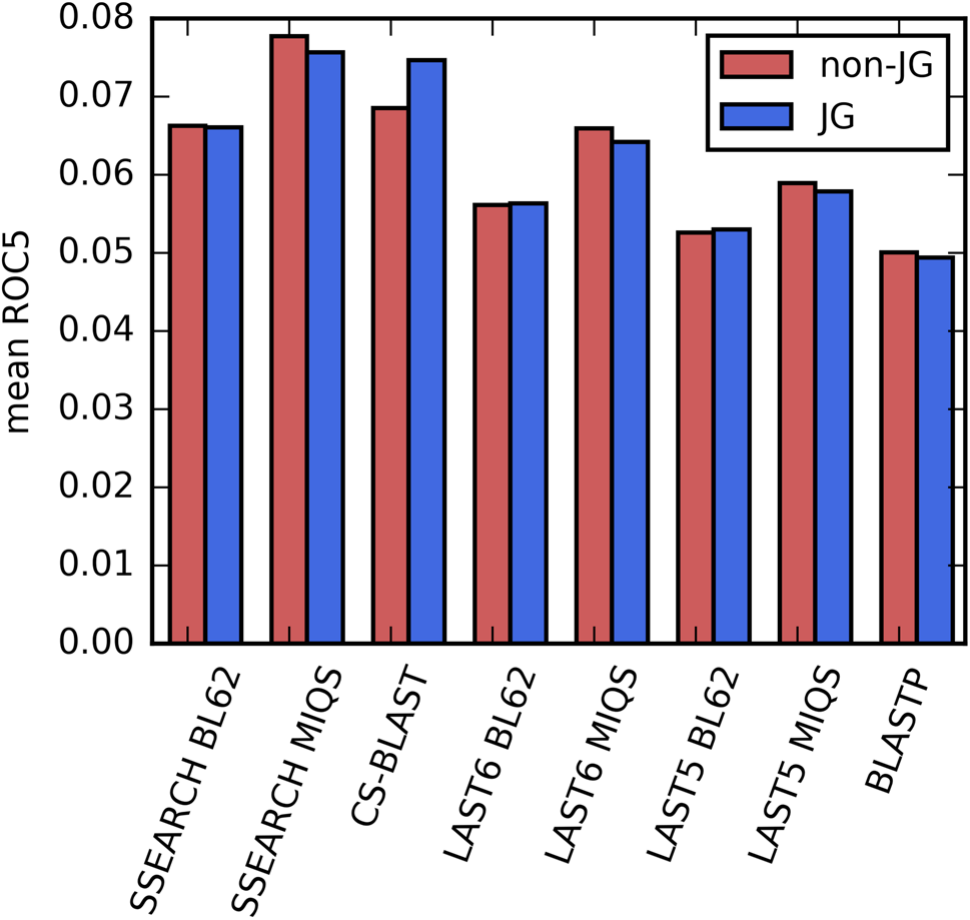
Homology detection benchmark per query. Superfamily level homology detection performances are shown for all-against-all search of the SCOP20 validation set. Mean ROC_5_ scores for TPs and FPs collected until FDR = 10% in the ROC curve (Fig. 1) are shown. ‘JG’: some FPs are ignored according to the JG standard. See “Results” section for additional details

We also assessed the detection performances using the ROC_n_ score, which is defined as [24]

$$RO{{C}_{n}}=\frac{1}{nT}\sum\limits_{i=1}^{n}{{{t}_{i}},}$$

where *T* is the total TP count and *t*_*i*_ is the TP count until the *i*-th FP appears. The obtained FPs can be less than 5, in which case, the unobserved hits are regarded as FPs. The ROC_5_ score therefore is “the normalized area under the ROC curve until the fifth FP” [22]. Mean ROC_5_ scores calculated using TPs and FPs retrieved until FDR = 10% in the ROC analysis (Fig. 1) are shown in Fig. 2. The ROC_5_ result shows good agreement with Fig. 1, demonstrating the superiority of LAST5 and LAST6 with MIQS over BLASTP, and the comparative performance of LAST6 using MIQS with SSEARCH using BLOSUM62. It is also readily apparent that CS-BLAST is extremely sensitive to application of the JG standard. The performance of SSEARCH using MIQS is comparable to that of CS-BLAST in the JG standard and is better in the non-JG standard.

**Fig. 3:**
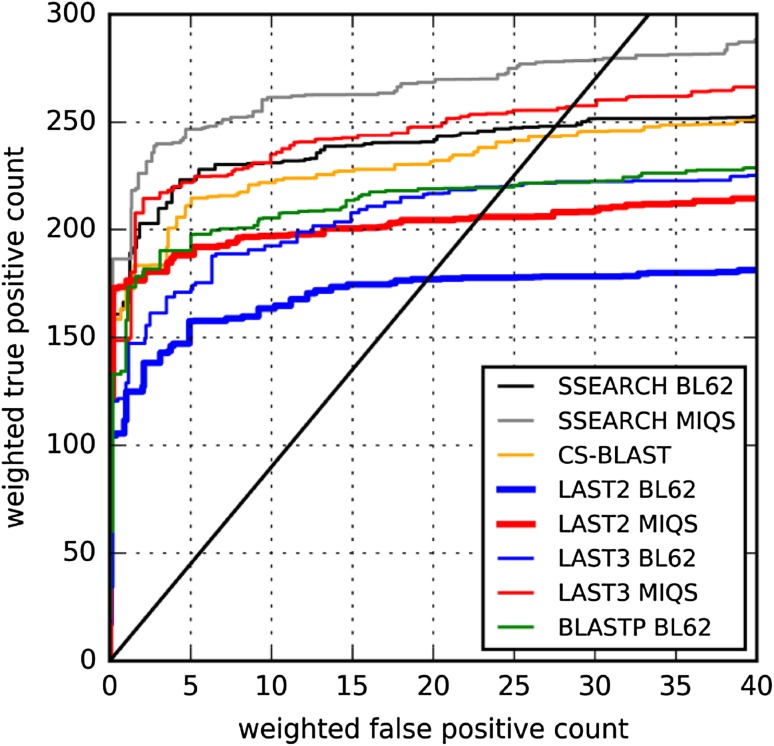
Superfamily level homology detection benchmark across database searches of CATH20-SCOP versus CATH20-SCOP. ROC plot for weighted FP versus weighted TP counts up to particular E-values. Each FP or TP is weighted by 1/(number of other domains in the query superfamily). The *solid black line* represents FDR = 10%. See “Results” section for additional details

We then confirmed the robustness of the results described above, by using CATH20-SCOP, which is regarded as independent of the SCOP 1.75 release. Figure 3 presents results of all-against-all searches with CATH20-SCOP. Because of the database size difference, LAST performance against CATH20-SCOP saturates earlier than that against UniRef50+ approximately at *m* = 10^3^. The ROC curve trends resemble the curves for *SCOP20 validation* (Fig. 1), indicating that LAST with MIQS is as sensitive as CS-BLAST and SEARCH with BLOSUM62.

### Alignment quality comparison

Alignment quality is another important factor to be considered in the selection of database search methods. Alignment quality is crucially important for downstream modeling such as protein structure prediction [25, 26]. We therefore examine the alignment qualities of database search methods using the previously established 588 pairwise DaliLite alignments of CATH20-SCOP benchmark set. DaliLite aligns two sequences based on structural information. Therefore, it is much more precise than pairwise aligners, which rely solely on sequences. We compared sequence alignments generated using each method with the structural alignments generated by DaliLite as reference alignments, and evaluated the alignment quality of each method using two terms: sensitivity and precision of alignments. The alignment sensitivity, the ratio of correctly aligned residue pairs to structurally equivalent residue pairs, is defined as (N∩S)/S, where N is the number of residue pairs in the sequence alignment generated by each method and S is the number of residue pairs in the DaliLite alignment. The alignment precision, which is the ratio of correctly aligned pairs to aligned pairs, is defined as (N∩S)/N. For a given alignment output consisting of multiple hits for a single target protein, only the one with the greatest significance (with the lowest E-value) is used. Like the ROC analysis for the homology detection benchmark, the curve for the sum of sensitivity versus the sum of (1—precision) up to different E-value thresholds enables the evaluation of alignment sensitivity and precision, which share a tradeoff relation in the same space. This mode of comparison is more effective than separate evaluation of sensitivity and precision.

**Fig. 4:**
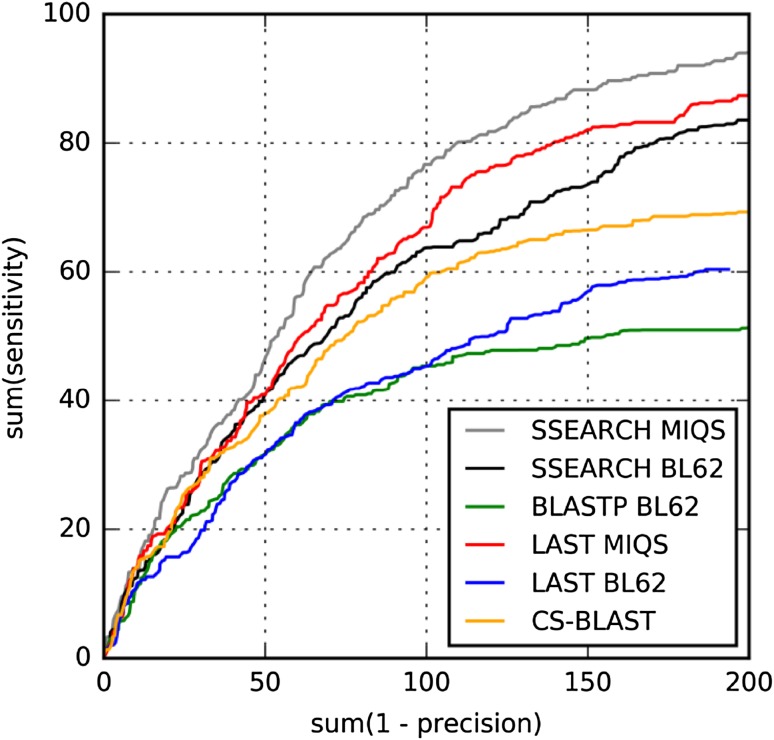
Alignment quality benchmark for pairwise alignments (*n* = 588) constructed using sequences in the CATH20-SCOP set. ROC plot for the sum of sensitivity against the sum of (1—precision) until varying E-values is shown across all pairwise alignments, where sensitivity = TP/(TP + FN) and precision = TP/(TP + FP)

Figure 4 shows that LAST with *m* = 10^4^ and BLASTP with BLOSUM62 have similar degrees of alignment quality. SSEARCH and CS-BLAST are significantly better than BLASTP and LAST with BLOSUM62. Remarkably, MIQS yields immense performance improvement in LAST, even exceeding those of SSEARCH with BLOSUM62 and CS-BLAST. The improvement by MIQS is also considerable for SSEARCH, underscoring its robustness.

### Computational resource usage comparison

**Fig. 5:**
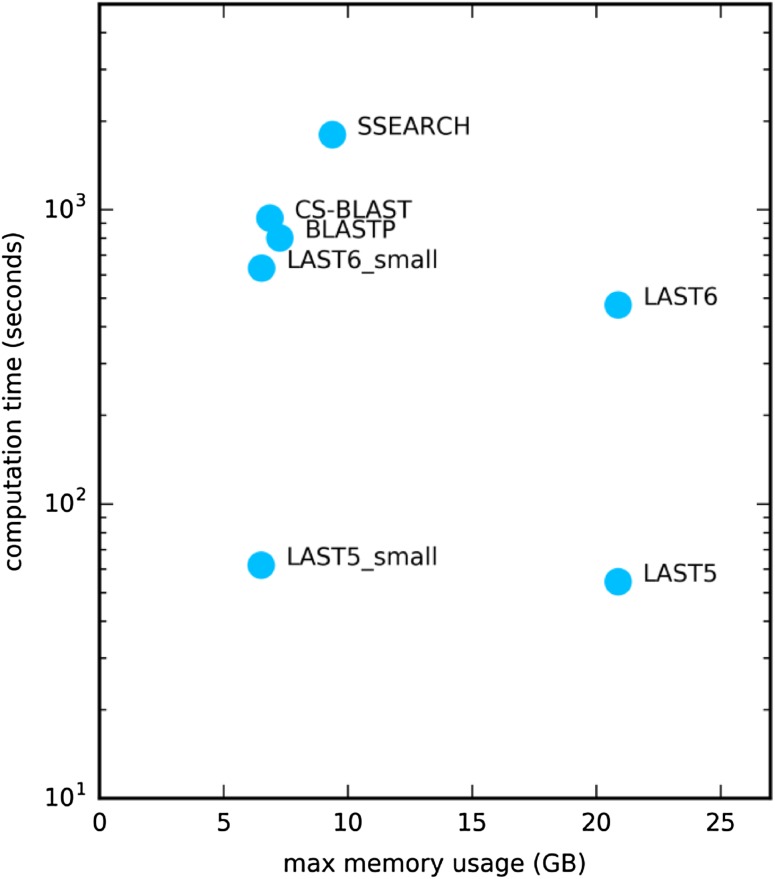
Running time and maximum memory usage of ten searches against UniRef50+. Time (s) is shown in a log10 scale. ‘LASTn_small’: the UniRef50+ database for LAST was constructed with ‘−s 7G’ option, so that the LAST search occupies less than 7G of memory

Because publicly available genetic data are increasing exponentially, database search method speeds are becoming increasingly important. To assess computational resource usage by the database search methods, ten sequences chosen randomly from *SCOP20 validation* were submitted as a query in a multi-fasta format file against UniRef50+ using database search methods with BLOSUM62 if applicable. Figure 5 shows that LAST becomes slower as *m* increases. LAST5 and LAST6 are 14.7 and 1.7 times faster than BLASTP, respectively, again indicating LAST’s dominance. LAST6 are, respectively, 2.0 and 3.8 times faster than CS-BLAST and SSEARCH. Given the high detection and alignment performance (Figs. 1, 2, 3, 4), LAST6 with MIQS is a more time-efficient method than either CS-BLAST or SSEARCH.

The higher speed of LAST might be attributable in part to its intensive memory usage because LAST requires much more memory than other methods do (Fig. 5). Actually, LAST requires more than 20 GB of memory for the database search of UniRef50+, which is more than two times that of other methods. We can restrict LAST’s memory usage to 7 GB (‘−s 7G’ option for *lastdb* command), which is a similar amount of memory usage to those of CS-BLAST and SSEARCH, by constructing smaller sub-databases, which makes LAST slightly slower, but still faster than competitors, indicating its resource effectiveness (Fig. 5). It is noteworthy that numerous other alternatives are available to tune LAST performance (http://last.cbrc.jp/doc/last-tuning.html).

## Discussion

A substitution matrix governs proper alignment extension from the seed, affecting homology detection sensitivity. In our previous study [13], MIQS, which was optimized to robustly represent the known protein space of the SCOP database, was able to enhance homology detection performance, where SSEARCH (rigorous dynamic programming) was used for both the optimization and the performance evaluation. In this study we show that the application of MIQS also robustly improves homology detection performance of the seed-and-extend heuristic method (LAST), compared to BLOSUM62, using the SCOP20 validation set and its expansion, UniRef50+ with two different definitions of homology, and CATH20-SCOP, an independent benchmark. Fortunately, LAST allows new scoring schemes for such as MIQS. In contrast, BLAST is applicable only for a limited set of predefined scoring schemes: this is presumably because it cannot calculate statistical significance (E-values), without hard-coded, pre-calculated parameters for each scoring scheme that it does allow. LAST uses the ALP library to calculate E-values for any scoring scheme [27].

As shown in our previous work [13], seed-and-extend heuristic methods, such as BLAST and LAST, tend to produce short alignments, and so do substitution matrices based on protein blocks instead of alignments, such as the BLOSUM series. In contrast, MIQS tends to produce well balanced alignments, in terms of both alignment sensitivity and precision, compared to existing matrices, leading to improved alignment quality, as shown for SSEARCH and LAST. Note that the gap costs used in this study for LAST are suitable for preventing homologous over-extension (HOE), according to the estimates by the ALP library.

Both BLAST and LAST reduce computational costs by the seed-and-extend heuristic method, where the number of seeds primarily regulates the tradeoff between sensitivity and computational cost (time). Using LAST one can regulate the tradeoff by adjusting the *m* parameter to the size of database, as shown in this study. LAST with *m* = 10^5^, for instance, works 20 times faster than BLAST against a database consisting of around 15 million sequences while maintaining BLASTP-level sensitivity. This demonstrates that LAST’s adaptive seeding based on the seed-frequency statistics greatly overwhelms BLAST’s fixed-length seeding for remote protein homolog search. With MIQS, LAST with *m* = 10^6^ can achieve database searches that are as sensitive as those of CS-BLAST and SSEARCH about two and four times faster, respectively, demonstrating that combining the heuristic method, LAST, with a sensitive matrix, MIQS, is a time-efficient alternative for remote homology search.

## Electronic supplementary material

Below is the link to the electronic supplementary material.

Supplementary material 1 (DOCX 209 KB)

## Abbreviations

ROC: Receiver operating characteristic
FDR: False discovery rate
TP: True positive
FP: False positive
