# Supplementary material for: Protein sequence-similarity search acceleration using a heuristic algorithm with a sensitive matrix
Source: J Struct Funct Genomics. 2017 Jan 12;17(4):147–54. doi: 10.1007/s10969-016-9210-4 (PMC5274646; doi:10.1007/s10969-016-9210-4)

Figure S1. Superfamily-level homology detection benchmark across database searches of the SCOP20 validation sequences against SCOP20 validation. ROC plot for weighted FP versus weighted TP counts up to particular E-values. Each FP or TP is weighted by 1/(the number of the other domains in the query's superfamily). Some FPs are ignored according to the JG standard in (B) but not in (A). Solid black line indicates FDR = 10%. See Results for details.


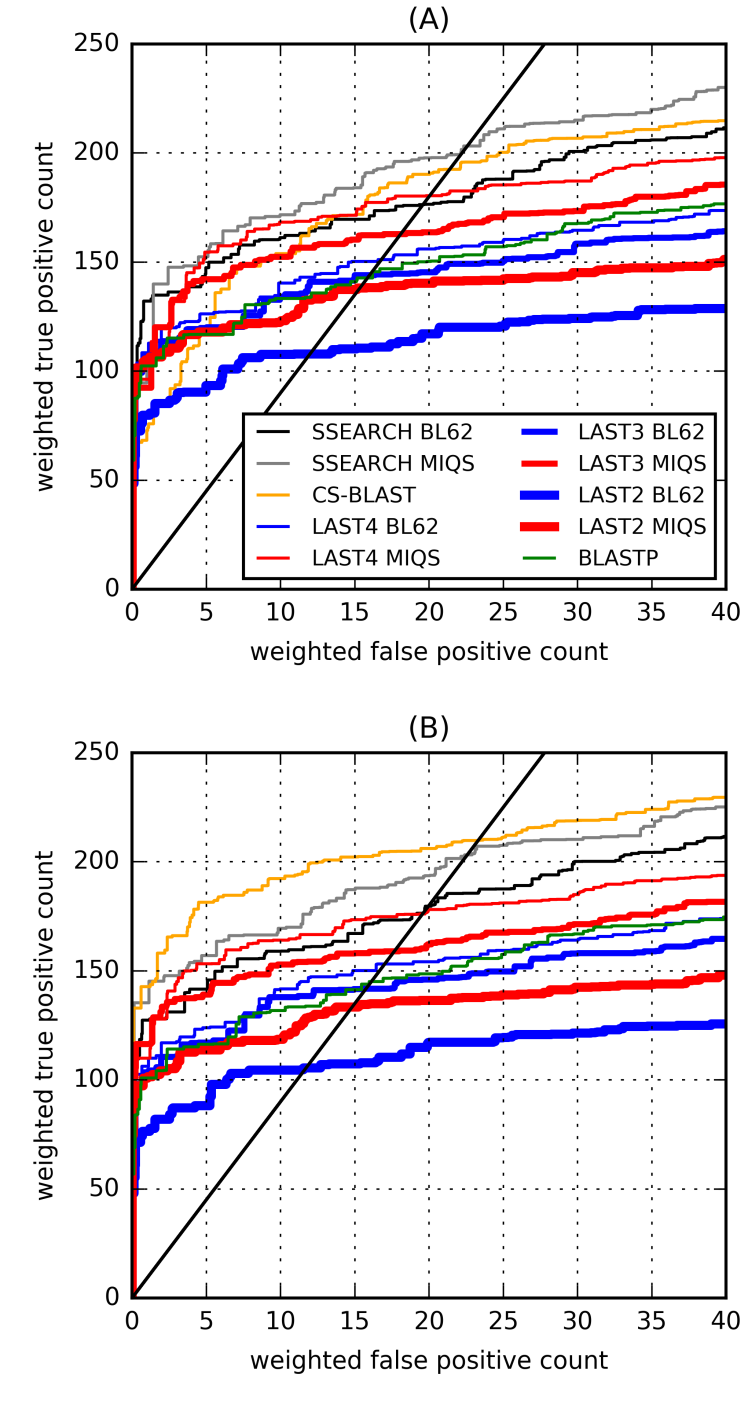

Supplement: Supplementary file 1 — Supplementary material 1 (DOCX 209 KB) [file 10969_2016_9210_MOESM1_ESM.docx]
